# The bone microenvironment promotes tumor growth and tissue perfusion compared with striated muscle in a preclinical model of prostate cancer in vivo

**DOI:** 10.1186/s12885-018-4905-5

**Published:** 2018-10-16

**Authors:** Haider Mussawy, Lennart Viezens, Malte Schroeder, Svenja Hettenhausen, Jördis Sündermann, Jasmin Wellbrock, Kai Kossow, Christian Schaefer

**Affiliations:** 1 Department of Orthopaedic Surgery, University Medical Center Hamburg-Eppendorf, 20246 Hamburg, Germany; 2 Department of Trauma, Orthopaedic, and Plastic Surgery, University Medical Center Goettingen, Goettingen, Germany; 3 Department of Spine Surgery, Klinikum Bad Bramstedt, 24576 Bad Bramstedt, Germany; 4 Department of Hematology, Oncology and Stem Cell Transplantation with Section Pneumology, University Medical Center Hamburg-Eppendorf, 20246 Hamburg, Germany; 5 Center of Psychosocial Medicine, Institute and Policlinics of Medical Psychology, University Medical Center Hamburg-Eppendorf, 20246 Hamburg, Germany

**Keywords:** Bone microenvironment, Femur window, Dorsal skinfold chamber, Tumor growth, Prostate cancer, Intravital microscopy

## Abstract

**Background:**

Prostate cancer-related morbidity is associated with its preferential spread to the bone. Although the molecular interactions between the bone microenvironment and cancer cells have been researched extensively, the relevance of the microvascular properties of prostate cancer bone metastases remains largely unknown. Most preclinical studies focusing on microvascular analyses are based on heterotopic tumor implantation, whereas the impact of the microenvironment on site-specific growth behavior and angiogenesis is rarely addressed.

**Methods:**

The microvascular changes associated with tumor growth in bone and soft tissue were characterized by implanting single cell suspensions of LnCap, Du145, and Pc3 cells into the femur (femur window) or striated muscle (dorsal skinfold chamber) of NSG mice. Tumor growth and the local microvasculature were analyzed for 21 days using intravital fluorescence microscopy.

**Results:**

The results showed a higher engraftment of tumor cells in bone than in striated muscle associated with accelerated growth of LnCap cells and Pc3 cells. Permeability, blood flow, and tissue perfusion rates were greater in bone than in striated muscle. Du145 cells showed similar growth behavior in both tissues with similar vascular properties. The bone microenvironment facilitated tumor engraftment and growth. Increased microvascular density in striated muscle led to a higher tumor burden during early growth, whereas the increased perfusion promoted later prostate cancer growth in bone.

**Conclusions:**

Monitoring prostate cancer microcirculation in bone and soft tissue may be useful to evaluate the organ-specific efficacy of new treatments.

**Electronic supplementary material:**

The online version of this article (10.1186/s12885-018-4905-5) contains supplementary material, which is available to authorized users.

## Background

Prostate cancer is the most common cancer in men and the sixth leading cause of cancer-related death among men worldwide [1]. It originates in soft tissues and is a relatively slow-growing tumor; however, it has a high probability of forming metastases in the skeleton, which results in significant disease morbidity and mortality including intractable bone pain and pathological fractures. Bone tissue is the preferred metastatic site and provides a supportive microenvironment where prostate cancer cells can reside and grow [2]. Despite the known impact of the local microenvironment and site-specific microvascular properties on tumor progression, relatively little is known about the microcirculation of bone metastases [3, 4]. This can be largely attributed to the limited availability of suitable preclinical models [5], especially the difficulties in generating mouse models of bone metastasis [6], and limitations associated with imaging of bone tissue at a high spatial resolution [7]. Heterotopic tumor implantation in soft tissues is commonly used to characterize tumor microcirculation, growth, and susceptibility to anti-angiogenic therapies; however, the influence of the host tissue microenvironment on tumor characteristics is rarely addressed [3, 6, 8–13].

Hence, we developed a bone tumor model that allows the continuous observation of tumor microvascular properties and growth in vivo, and described morphological angiogenic alterations during tumor growth in bone [7, 14–16].

To determine the effect of the microvasculature of prostate cancer growing in bone and striated muscle on growth behavior, the prostate cancer cell lines LnCap, Du145, and Pc3 were implanted into the femur [femur window (FW)] and striated muscle [dorsal skinfold chamber (DSC)] of non-obese diabetic/severe combined immunodeficiency/y-chain [NOD-Prkds IL2rg (NSG)] mice. After implantation of the cancer cells, the local microcirculation was analyzed for 21 days by intravital fluorescence microscopy to determine the effect of the environment on microvascular properties during tumor growth in bone and in striated muscle.

## Methods

### Cell lines

The prostate cancer cell lines LnCap, Du145, and Pc3 were transfected with the fusion protein mCherry, a derivative of the red fluorescent protein, using Lipofectamine (Invitrogen, Karlsruhe, Germany). Cells with a strong red signal were selected by fluorescence-activated cell sorting (> 95% expression; FACSAriaII, BD Biosciences, Heidelberg, Germany). Cells were grown in D-MEM/F12 medium containing 10% fetal bovine serum. The cells were cultured at 37 °C and 5% CO_2_ in a humidified incubator. The cell lines PC3 (catalogue number ACC-465), Du-145 (catalogue number ACC-261) and LnCap (catalogue number ACC-256) were authenticated at DSMZ (Deutsche Sammlung von Mikroorganismen und Zellkulturen GmbH) during the term of experiments. The cell lines were routinely tested for mycoplasma contamination with MycoAlert Mycoplasma Detection Kit from Lonza.

### Tumor model

Male NSG mice (12–14 weeks old) (University Medical Center Hamburg-Eppendorf, Germany) were used in the study. Animals were kept in a 12:12 h light:dark cycle at 24 °C and 50% humidity. Mice were caged individually and had free access to tap water and standard pellet food (Altromin, Lage, Germany). The study was approved by the local governmental animal care committee (protocol number 05/12) and was conducted in accordance with the German legislation on the protection of animals and the National Institutes of Health (NIH) Guidelines for Care and Use of Laboratory Animals (NIH Publication #85–23 Rev. 1985). All surgical procedures were performed under aseptic conditions while maintaining body temperature at physiological levels using a heating pad (Omnilab PST 100, Jürgens, Germany). Prior to surgical procedures, mice were anesthetized (7.5 mg of ketamine hydrochloride and 2.5 mg of xylazine/100 g of body weight), and the skin surrounding the site of the surgical approach was shaved and depilated.

### Preparation of the dorsal skinfold chamber

The microcirculation and tumor growth in striated muscle tissue were analyzed in the DSC. The chamber preparation was described previously in detail [17]. Prior to the final DSC fixation, a suspension of 1 × 10^6^ cells in 1 ml of phosphate buffered saline was centrifuged, and after removing the supernatant, LnCap, Du145, or Pc3 cells were implanted into the striated muscle tissue of NSG mice.

### Preparation of the femur window

The microcirculation and tumor growth in bone were analyzed in the FW. Chamber preparation was described previously in detail [7]. The aseptic surgical conditions and use of heating plates during the operation were as described above. Prior to the final FW fixation, a suspension of 1 × 10^6^ cells in 1 ml of phosphate buffered saline was centrifuged, and after removing the supernatant, LnCap, Du145, or Pc3 cells were implanted into the cancellous bone of the diaphysis of NSG mice.

### Experimental protocol

The animals were randomly divided into six groups (*n* = 105): three groups had FW implantation and three groups had DSC implantation, each with LnCap, Du145, or Pc3 cells. Each group had 15 FWs or 20 DSCs implanted. Animals without tumor engraftment on day 7 or with clinical signs of postoperative infection were excluded. Mice with excessive tumor growth and with subsequent femur fracture were sacrificed with an overdose of the anesthetic administered via the tail vein. Intravital fluorescence microscopic analysis of tumor growth, vascularization, and effective vascular permeability (PERM) was performed weekly after chamber implantation. At the end of the in vivo experiments, the animals were sacrificed with an overdose of the anesthetic.

### Intravital fluorescence microscopy

To obtain microcirculatory parameters, three locations within the tumor were analyzed (Additional file 1: Figure S1 and Additional file 2: Figure S2) using an intravital fluorescence microscope (Axioplan, Zeiss, Oberkochen, Germany) and a 20× objective (LD Achroplan 20x/0.40, Zeiss). The microscope was equipped with fluorescence filter sets for fluorescein isothiocyanate (FITC) and red fluorescence protein, an intensified charge coupled device video camera (C-0377-1, Hamamatsu Photonics, Hamamatsu, Germany), a Photomultiplier Tube (R4632, Hamamatsu Photonics), and a Computer (Apple Power Macintosh, G4, Dual 500 MHz Power PC, 1 GB SDRAM) for digital signal recording and off-line analysis. During measurements, the body temperature was maintained at physiological levels using a heating pad. To eliminate movements of the transparent chamber caused by breathing, the chamber was fixed to the microscope using a custom-made clamp.

### Tumor growth and microcirculatory analysis

**Fig. 1:**
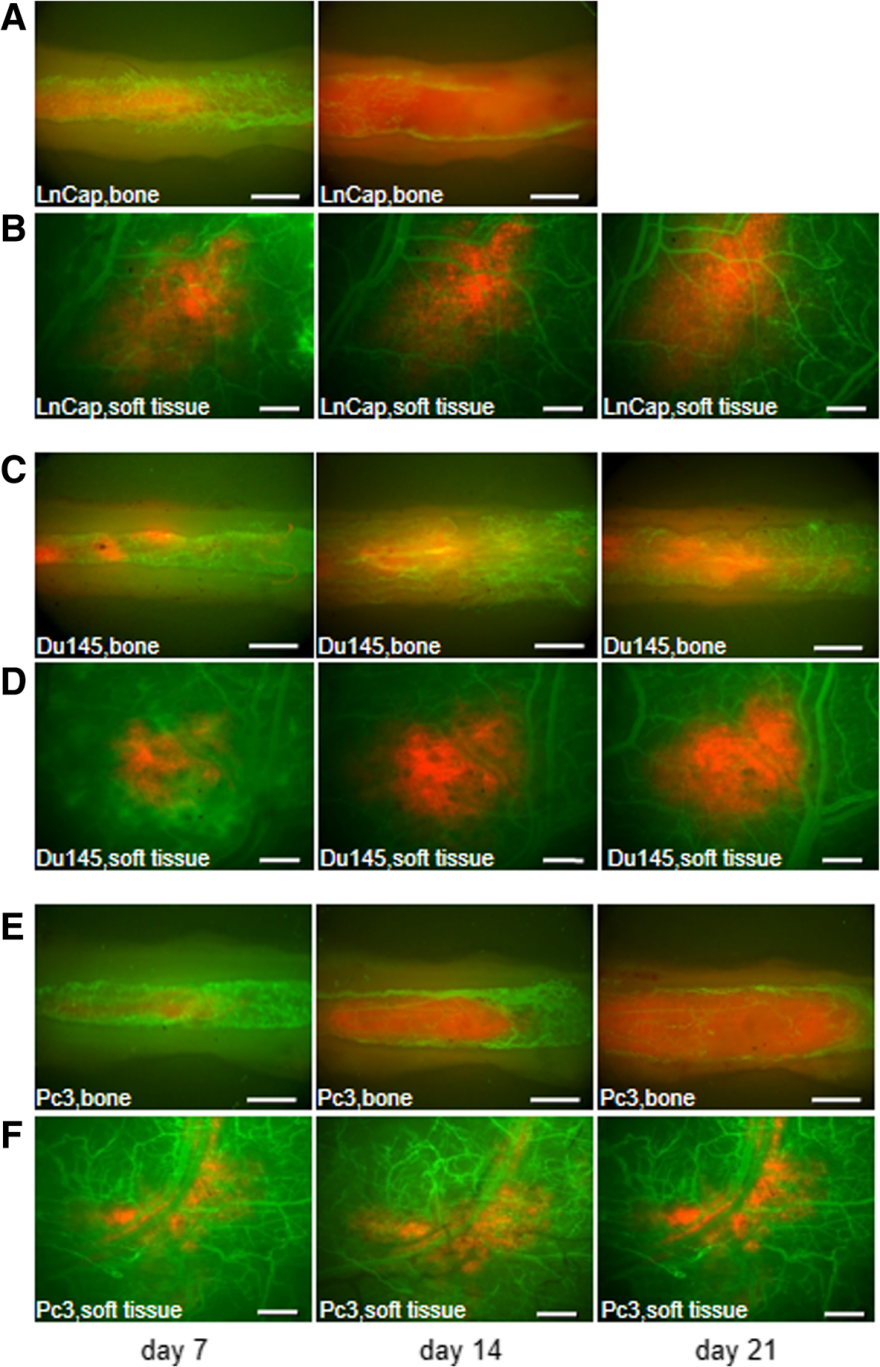
Intravital fluorescence microscopy of tumor growth and local microvessels in the femur window (**a**, **c**, **e**) with a 2.5× objective and in the dorsal skinfold chamber (**b**, **d**, **f**) with a 1.25× objective after contrast enhancement with 0.1 ml of 5% FITC-labeled dextran 150,000 on days 7, 14, and 21. Note the different tumor growth rates of LnCap (**a**, **b**), Du145 (**c**, **d**), and Pc3 (**e**, **f**) cells (scale bars **a**, **c**, **e** = 500 μm; **b**, **d**, **f** = 680 μm)

Assessment of tumor growth was performed with epi-illumination. Analysis of the tumor area was performed using Axiovison software (Axiovison 4.6, Carl Zeiss Jena GmbH, Jena, Germany; Fig. 1a–f). For contrast enhancement of the microcirculation, 0.1 ml of 5% FITC-labeled dextran 150,000 (Molecular Probes, Invitrogen Ltd., Paisley, UK) was administered via the tail vein. Fluorescence images were recorded digitally and non-compressed for 10 s, and analyzed off-line using the software package from the NIH (NIH Image 1.62). Functional capillary density (VD), i.e., the length of all perfused microvessels per observation area, was measured and expressed in cm/cm^2^. Mean diameters (D), centerline velocity (Vmean), and blood flow rate (BFR) were measured in all perfused microvessels. D was measured in μm perpendicularly to the vessel path. Vmean was analyzed using NIH Image 1.62. BFR was calculated using the formula Q = π x (d/2)^2^ x v / 1.6 [pl/s], where 1.6 represents the Baker-Wayland factor [18] to correct for the parabolic velocity profile in microvessels. The tissue perfusion rate (TPR), i.e., blood flow rate per time and area, was obtained using VD and BFR as described previously [19].

### Effective vascular permeability

PERM was measured as described previously [18]. Briefly, after the application of 0.1 ml of 5% FITC coupled to bovine serum albumin via the tail vein, the fluorescence intensity was measured intermittently for 10 min and recorded digitally (PowerLab/200 AD Instruments Pty Ltd., Castle Hill, Australia). The permeability value was calculated as P = (1 - HT) V/S (I0 - Ib) x dI/dt + 1/K), where I is the average fluorescence intensity of the whole image, I0 is the value of I immediately after filling of all vessels by FITC-BSA, and Ib is the background fluorescence intensity. The average hematocrit (HT) of vessels is assumed to be equal to 19% [20]. V and S are the total volume and surface area of vessels within the tissue volume covered by the surface image. The time constant of BSA plasma clearance (K) is 9.1 × 10^3^ s [21].

### Statistics

Differences between the study groups were analyzed with SPSS (IBM SPSS Statistics 19, Chicago, IL, USA) using the one-way repeated measures ANOVA F-test (Greenhouse Geisser adjustment in case of violated assumption of sphericity) between different measurement points. A post-test (Bonferroni) were conducted to determine significance between individual time points once the significance of the overall test was determined. All values are expressed as mean ± SD. Statistical significance was based on *p*-values < 0.05.

## Results

Microvascular alterations during prostate cancer growth were analyzed in vivo in striated muscle and in bone using intravital fluorescence microscopic analysis of the DSC and FW of mice implanted with three different cell lines.

### Tumor take rate and growth behavior

**Fig. 2:**
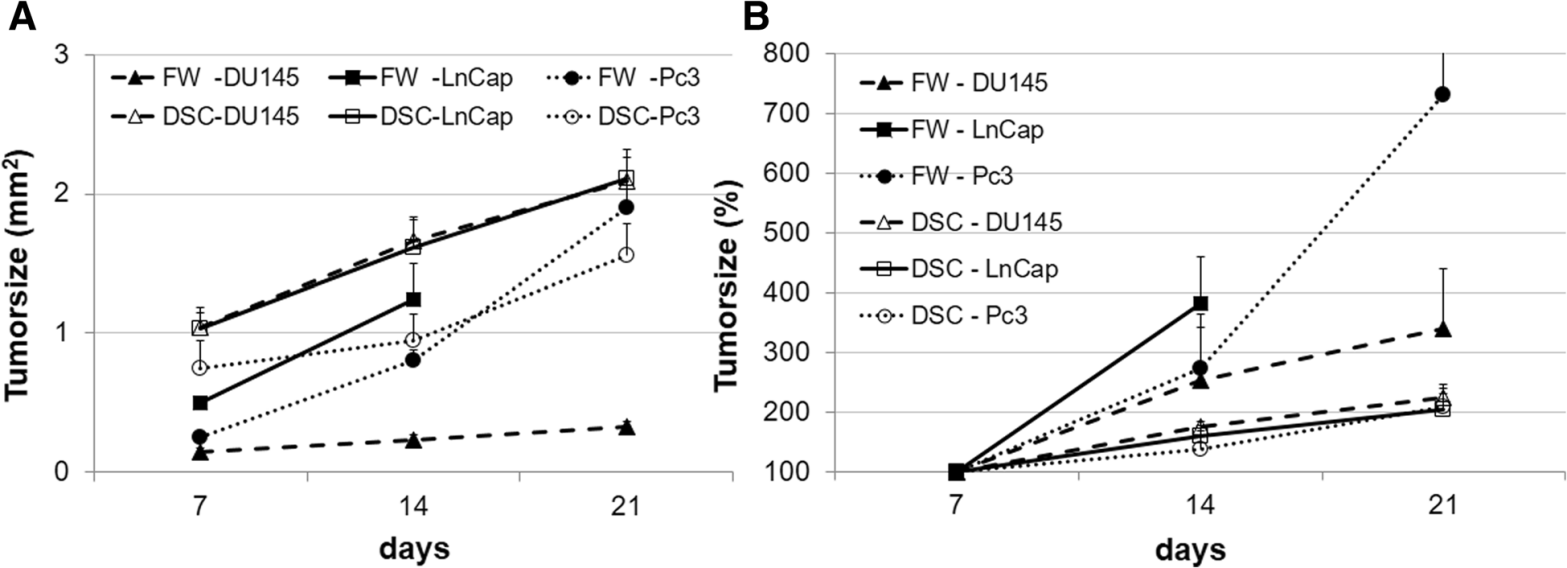
Tumor growth in the femur window (FW) and dorsal skinfold chamber (DSC) during 21 days as absolute (**a**) and relative (**b**) values. The Fig. **a** shows the significant higher tumor burden of LnCap and Du145 cells in striated muscle than in bone. The tumor burden of Pc3 cells was higher in striated muscle than in bone, although the difference was not statistically significant. In Fig. **b** the growth of Pc3 cell-derived tumors was significantly greater in bone than in striated muscle on days 14 and 21. LnCap cells showed greater tumor growth in bone than in striated muscle tissue on day 14. The growth of Du145 cells showed no significant differences during the observation period

The take rate was lower in striated muscle than in bone tissue. Engraftment of LnCap and Du145 cells was equal with 70% and 93% in striated muscle and bone, respectively. Similar rate differences were observed in Pc3 cells at 45% vs. 67% in striated muscle vs. bone. Tumor size on day 7 was 2-fold greater in striated muscle than their counterparts in bone (Additional file 3: Table S1 and Fig. 2a). Tumors in both tissues showed significant growth at 7–21 days (Figs. 1 and 2 and Additional file 3: Table S1). The Pc3 cells demonstrated similar trends albeit without achieving statistical significance. Tumors in striated muscles exhibited similar growth from 7 (defined with 100%) to 21 days without statistically significant differences between cell lines (LnCap, 205%, *n* = 12; Du145, 225%, *n* = 10; and Pc3, 210%, *n* = 4); however, in bone, LnCap- and Pc3-derived tumors showed significant variation in growth behavior, with a nearly quadruplication and triplication time of 14 days in LnCap (382%) and Pc3 (274%), respectively. This caused femur fractures in all LnCap mice after day 14, and mice were euthanized in accordance to the study protocol. The rapid growth of Pc3-derived tumors resulted in tumor areas of 730% on day 21, with femur fractures occurring thereafter (Figs. 1 and 2b); these mice were euthanized. Du145 cell-derived tumors showed variation in growth rate with a doubling time of 14 days (Du145, 250%, *n* = 14) and resulted in increased tumor area of 340% on day 21 in bone, compared to striated muscle tissue tumors with relative tumor growth of 225%.

### Tumor microcirculation depends on the host tissue

**Fig. 3:**
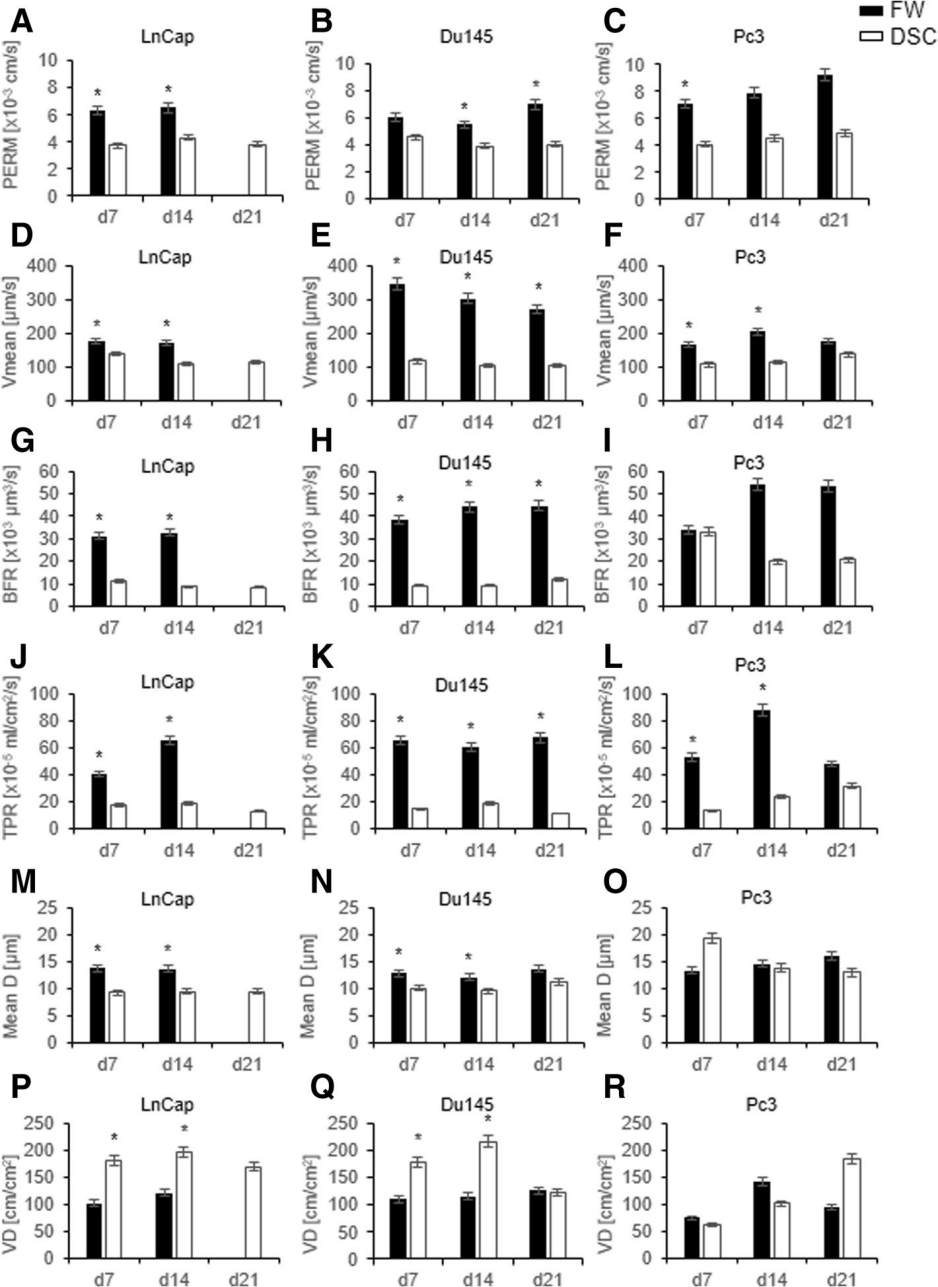
**a**–**r** Microvascular parameters on days 7, 14, and 21 after tumor implantation measured in three regions of interest (two in the border zone and one in the center zone of the tumor) of LnCap, Du145, or Pc3 cells in the dorsal skinfold chamber (DSC) and in the femur window (FW) as assessed by intravital fluorescence microscopy and computer-assisted image analysis. The black bars represent the FW group, and the white bars represent the DSC group. All values are presented as the mean ± SD. (* *p* < 0.05). *Abbreviations: PERM, effective vascular permeability; Vmean, centerline velocity; BFR, blood perfusion rate; TPR, tissue perfusion rate; Mean D, mean diameter; VD, vessel density*

The bone microenvironment was associated with increased microvascular permeability, blood flow, and tissue perfusion in comparison to striated muscle (Fig. 3a–c, g–i, and j–l). The functional vascular density was higher in the DSC than in the FW for LnCap and Du145 tumors on days 7 and 14, whereas the differences did not reach significance for Pc3 tumors because of the small sample size in the DSC (Fig. 3p–r). The Vmean and D values were higher in bone than in striated muscle in all cell lines (Fig. 3d–f and m–o). The Vmean was highest for Du145 cells in bone compared with the other cell line-derived tumors in bone and striated muscle (Fig. 3d–f).

## Discussion

We herein demonstrate the significant impact of the bone and soft tissue microenvironments on prostate cancer growth and tumor microvascular properties in vivo during a period of 21 days by intravital microscopy of the FW and DSC.

### Advantages and limitations of the tumor model

In recent years, many indirect observation methods were developed to analyze tumor growth in bone and the morphological and functional aspects of angiogenesis. Because rodents do not commonly form spontaneous bone metastases, designing a research model is difficult [22]. Xenograft models generated by implantation of human tumor cells into immunodeficient mice considerably improved our understanding of the tumor microenvironment [23–25]. But the results obtained on immunodeficient mice cannot be directly translated onto the human biology. Experimental metastases are commonly generated by intracardial or intratibial injection of cancer cells to induce bone metastasis [26, 27]. Although intracardial injection mimics the process of metastasis, the site of bone metastases and the time of development cannot be controlled in the study design. Another frequently used procedure is the subcutaneous application of tumor cells and subsequent postmortem evaluation, which is useful for histological examination; however, similar to the previous model, this design does not permit continuous in vivo imaging of functional microvascular alterations during tumor growth [28]. Models that use direct inoculation of tumor cells into bone, as described in this study, mimic the final stages of bone colonization, whereas they do not address the proliferation of primary neoplasm, intravasation into blood vessels, extravasation into bone marrow, tumor cell dormancy, paracrine local tissue and activation of the tumor cells. In addition, the role of intact immune system as controlling the tumor growth can not be analyzed with the presented model [29]. Furthermore, the direct inoculation of a large number of tumor cells may generate a different initial tumor size. The difference in tumor size is a weakness in this study since tumor microcirculation may vary with size of the tumor.

In addition to the difficulties associated with generating bone metastases in rodents within a defined time frame and at a specific growth site, monitoring tumor growth and angiogenesis in bone is difficult. Bauerle et al. investigated bone metastasis in nude rats using magnetic resonance imaging, volumetric computed tomography, and ultrasonography [30]. These technologies can be used to assess tumor growth and detect solid metastases; however, these methods are limited regarding the detection of tumors at the early stages of formation or the visualization of functional microvascular changes associated with tumor growth in vivo in real time [31]. Imaging technologies lack spatial resolution or the ability to monitor morphological and functional aspects of microcirculation during tumor growth. In this context, intravital microscopy using transparent chamber techniques is a successful approach to investigate the microcirculatory properties of various tissues at a high spatial resolution [7, 12, 13, 32–34].

Each model offers advantages and disadvantages, and no single ideal model exists. Intravital fluorescence microscopy provides anatomical and functional insight into tumor pathophysiology, including angiogenesis and the microenvironment in vivo, in a non-invasive and non-destructive manner [4].

### Bone tissue increases the take rate in the prostate cancer cell lines LnCap and Pc3 and is associated with reduced early tumor growth

A large body of literature indicates that the tumor microenvironment is crucial for tumor progression and the response to treatment [9]. The host tissue determines tumor cell survival and growth via molecular interactions [35]. Consistent with previous studies, we showed that the microenvironment affected the tumor take rate [36, 37]. The tumor take rate was approximately one third higher in bone tissue than in muscle for all three cell lines despite equal amounts of inoculated tumor cells. The cell lines used were suitable for experimental studies of bone metastasis with engraftment rates of over 66%. However, the use of Pc3 cells in xenograft DSC models in NSG mice is limited because of the low engraftment rates in striated muscle.

Despite the higher engraftment rates, the initial tumor size for the three cell lines was nearly 50% smaller in bone than in muscle on day 7 (Fig. 2a and Additional file 3: Table S1). In addition to paracrine survival factors, which influence take rate, the microcirculation of the host tissue has a major impact on tumor growth because the survival and growth of cells depends on an adequate supply of oxygen and nutrients [38]. Pre-existing host vessels support or limit early tumor growth before angiogenesis [39]. Since the diffusion of oxygen is limited to 150–200 μm [40], the higher functional vascular density and consequent decrease in intervascular distance in striated muscle may promote early tumor growth compared with that in bone tissue. These observations are in line with the microvascular architecture in the dorsal skinfold chamber that presents the usual striated muscle with regular microvessel anatomy [41]. In contrast, the blood supply in bone is guaranteed by fenestrated capillaries (longitudinal and transverse canals) [42, 43]. This system, normally allows the intravasation of developing myeloid cells into the blood due to the large pores present [44]. These pores (30–40 μm) may facilitate extravasation for tumor cells and therefore support tumor growth in bone.

### The microvascular properties of bone substantially promote tumor growth in LnCap and Pc3 tumors

Analysis of tumor size according to initial tumor area on day 7 demonstrated a pronounced growth rate in LnCap and Pc3 tumors in the bone microenvironment (Fig. 2b). The rapid tumor growth led to femur fractures after 14 days in LnCap and after 21 days in Pc3 mice. Despite the mixed osteolytic/osteoblastic pattern of LnCap tumors compared with the osteolytic growth of Pc3 cells, pathologic fractures were delayed in Pc3 tumors compared with those of LnCap cells [45]. This can be attributed to the difference in absolute tumor size, as the initial tumor burden was 50% lower in Pc3 tumors on day 7. Tumor growth was markedly lower in Du145 tumors than in those derived from other cell lines and corresponded to their counterparts grown in striated muscle tissue. The slow tumor growth in bone was consistent with previous observations [45].

Tumor growth beyond a few cubic millimeters is angiogenesis-dependent [46]. Angiogenesis is a prerequisite for tumor growth, whereas vessel density is not necessarily accompanied by tissue perfusion, and temporal variation during tumor growth needs to be assessed; therefore, intravital microscopy provides a detailed insight into tumor biology [34, 47, 48]. To determine whether the microvascular properties accounted for the differences in tumor growth between secondary bone and soft tissue tumors, we analyzed the functional and morphological aspects of tumor vessels.

Angiogenesis involves multiple interdependent steps, as described in previous studies [49]. One of the first steps is the degradation of the basement membrane, as indicated by increased PERM [50, 51]. Due to the vascular architecture in bone the effective vascular permeability was higher in the femur window than in the dorsal skinfold chamber. This may facilitate the first step in angiogenesis. Secondary bone tumors were associated with increased permeability compared with that in striated muscle tissue. This may promote prostate cancer growth in bone and contribute to impaired drug delivery via increased interstitial pressure [52, 53]. Knowledge of local bone vascularization, including PERM, could be essential for preventing the formation of secondary bone tumors in prostate cancer patients [54].

The lower vascular density of bone tumors, in particular LnCap and Du145 tumors, indicated that high vascularity is not necessarily associated with accelerated tumor growth, as microvessel density does not reflect the metabolic demand of a tumor [55]. Furthermore, we showed that, despite the lower vascular density, increased blood flow and vascular diameter resulted in increased tissue perfusion rates in bone tumors. Under physiological conditions, the tissue perfusion rate was similar between bone and striated muscle tissue in NSG mice, as previously demonstrated [56]. The different organ environments differentially affected tumor growth. The higher perfusion rate may have contributed to the accelerated tumor growth of Pc3 and LnCap tumors. The tumor size of Du145 was decreased. This could be associated with paracrine/autocrine mechanisms, which greatly contribute to tumor progression [57]. These paracrine factors may be crucial for the growth characteristics in both tissues. In bone, the osteocytes support cancer progression by cytokines production [58]. Furthermore, the osteoclasts are stimulated by tumor induced osteolytic factors (RANKL expression, Parathyroid hormone-related protein, Interleukin-6, matrix metalloproteinases and cathepsins). These factors induce (amongst others) the degradation of extracellular matrix and mineralized bone and increased invasion and migration of tumor cells [59].

The increased blood flow velocity of Du145 tumors, which was considerably greater than that of other cell lines and tissues, may have further contributed to this phenomenon because of impaired substrate exchange. The steady growth of Du145 tumors with high engraftment rates in bone and striated muscle could be used in subsequent studies to compare the effects of treatments for prostate cancer metastasis in both tissues.

## Conclusion

This preclinical model of prostate cancer provided insight into organ-related microcirculation and its possible impact on tumor growth in bone and striated muscle. Monitoring the in vivo responses of the tumor and microcirculation to new therapeutic agents may help elucidate the dependence of prostate cancer bone metastases on the microenvironment and contribute to the development of organ-optimized therapies.

## Additional files

Additional file 1: **Figure S1.** Three locations of interest within the Du145 tumor cells in soft tissue over observation period measured with intravital fluorescence microscope and a 20× objective. (TIF 368 kb)

Additional file 2: **Figure S2.** Three locations of interest within the Pc3 tumor cells in bone over observation period measured with intravital fluorescence microscope and a 20× objective. (TIF 287 kb)

Additional file 3: **Table S1.** Microcirculatory parameters and tumor growth in the femur window (FW) and dorsal skinfold chamber (DSC) during the observation period. (DOCX 33 kb)

## Abbreviations

BFR: Blood flow rate
D: Mean diameters
DSC: Dorsal skinfold chamber
FW: Femur window
HT: Average hematocrit
NSG: Non-obese diabetic/severe combined immunodeficiency/y-chain
PERM: Permeability
TPR: Tissue perfusion rate
VD: Vessel density
Vmean: Centerline velocity
