# Supplementary figures and images for: The bone microenvironment promotes tumor growth and tissue perfusion compared with striated muscle in a preclinical model of prostate cancer in vivo
Source: BMC Cancer. 2018 Oct 16;18:979. doi: 10.1186/s12885-018-4905-5 (PMC6192198; doi:10.1186/s12885-018-4905-5)

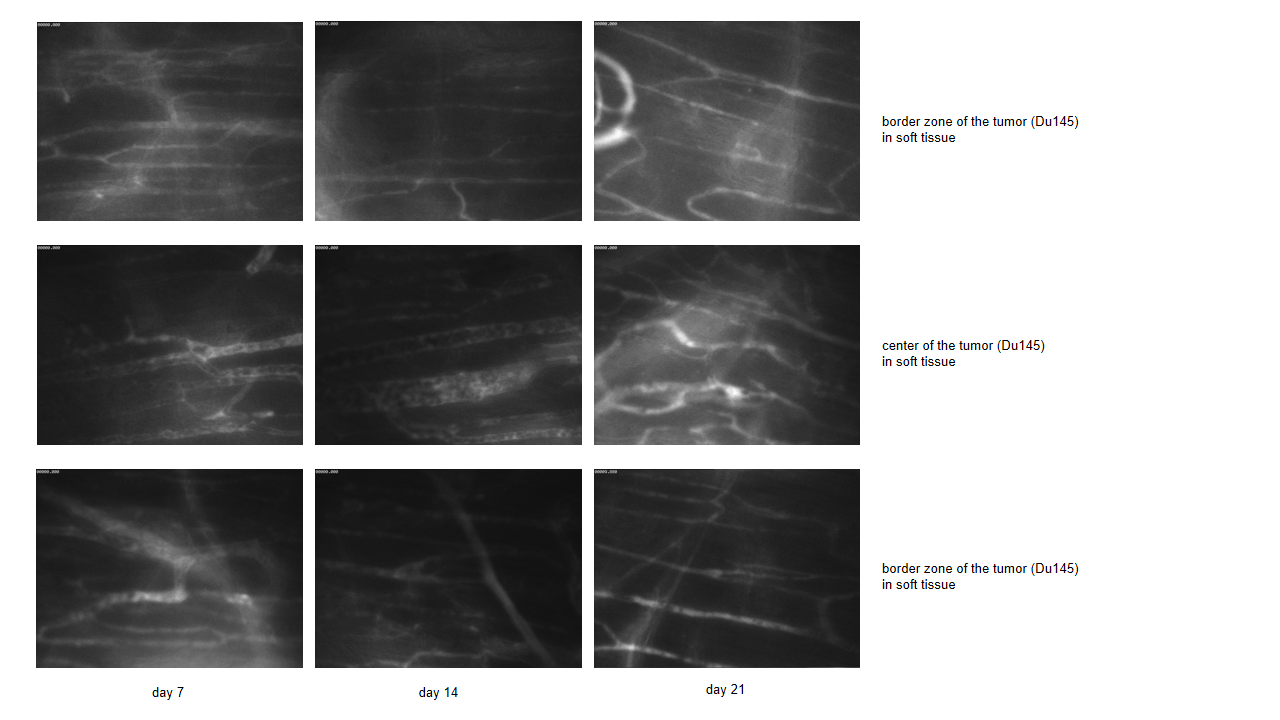

Supplement: Supplementary file 1 — Figure S1. Three locations of interest within the Du145 tumor cells in soft tissue over observation period measured with intravital fluorescence microscope and a 20× objective. (TIF 368 kb) [file 12885_2018_4905_MOESM1_ESM.tif]

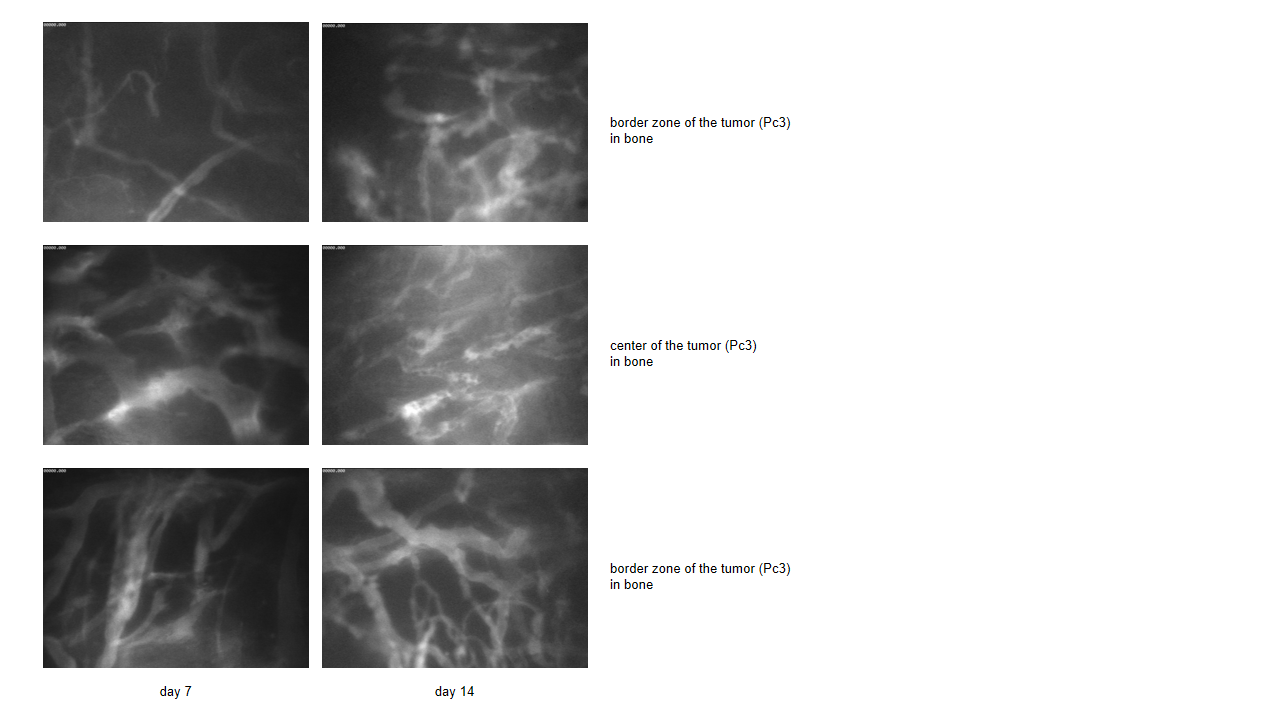

Supplement: Supplementary file 2 — Figure S2. Three locations of interest within the Pc3 tumor cells in bone over observation period measured with intravital fluorescence microscope and a 20× objective. (TIF 287 kb) [file 12885_2018_4905_MOESM2_ESM.tif]
