# Supplementary material for: The bone microenvironment promotes tumor growth and tissue perfusion compared with striated muscle in a preclinical model of prostate cancer in vivo
Source: BMC Cancer. 2018 Oct 16;18:979. doi: 10.1186/s12885-018-4905-5 (PMC6192198; doi:10.1186/s12885-018-4905-5)
Supplement: Supplementary file 3 — Table S1. Microcirculatory parameters and tumor growth in the femur window (FW) and dorsal skinfold chamber (DSC) during the observation period. (DOCX 33 kb) [file 12885_2018_4905_MOESM3_ESM.docx]

**Table S1.** Microcirculatory parameters and tumor growth in the femur window (FW) and dorsal skinfold chamber (DSC) during the observation period

| Parameter |  | Group | Day 7 | Day 14 | Day 21 |  |
| --- | --- | --- | --- | --- | --- | --- |
| Tumor growth | FW | Du145 | 0.15 ± 0.02 | 0.23 ± 0.34***** | 0.32 ± 0.04***^,^**** | F(2,26) = 32.920, p =.00, η^2^ =.717 |
| absolute (mm^2^) |  | LnCap | 0.36 ± 0.07 | 1.25 ± 0.26***** | - | F(1,4) = 19.587, p =.01, η^2^ =.830 |
|  |  | Pc3 | 0.29 ± 0.48 | 0.70 ± 0.71 | 1.89 ± 0.26****** | F(2,6) = 22.947, p =.00, η^2^ =.884 |
|  | DSC | Du145 | 1.02 ± 0.13 | 1.65 ± 0.23***** | 2.09 ± 0.17****** | F(2,18) = 34.314, p =.00, η^2^ =.792 |
|  |  | LnCap | 1.06 ± 0.17 | 1.64 ± 0.23***** | 2.11 ± 0.21****** | F(1.23,13.54) = 20.683, p =.00, η^2^ =.653 |
|  |  | Pc3 | 0.82 ± 0.45 | 1.02 ± 0.30 | 1.56 ± 0.22 | F(2,6) = 7.287, p=.02, η^2^ =.708 |
| Tumor growth | FW | Du145 | 1 | 2.53 ± 0.90 | 3.39 ± 1.02***** | F(1.04,13.58) = 4.672, p=0.4, η^2^ =.264 |
| relative to day 7 |  | LnCap | 1 | 3.82 ± 0.78***** |  | F(1.0,4.0) = 13.118, p=.02, η^2^ =.766 |
|  |  | Pc3 | 1 | 2.74 ± 0.66 | 7.32 ± 1.44****** | F(2,6) = 15.621, p=.00, η^2^ =.839 |
|  | DSC | Du145 | 1 | 1.76 ± 0.09***** | 2.24 ± 0.22****** | F(1.29,11.68) = 23.423, p=.00, η^2^ =.722 |
|  |  | LnCap | 1 | 1.61 ± 0.07***** | 2.05 ± 0.21****** | F(1.12,12.31) = 16.187, p=.00, η^2^ =.595 |
|  |  | Pc3 | 1 | 1.37 ± 0.41 | 2.09 ± 0.31 | F(2,6) = 6.234, p=.03, η^2^ =.675 |
| Mean diameter | FW | Du145 | 12.75 ± 0.84 | 12.00 ± 0.78 | 13.58 ± 0.91 | F(2,26) = .758, p=.47, η^2^ =.055 |
| (µm) |  | LnCap | 13.74 ± 1.17 | 13.57 ± 0.64 | - | F(1,4) =0.20, p=.89, η^2^ =.005 |
|  |  | Pc3 | 13.28 ± 0.82 | 14.58 ± 1.89 | 16.01 ± 2.09 | F(2,6) = .422, p=0.67, η^2^ =.123 |
|  | DSC | Du145 | 10.07 ± 0.75 | 9.40 ± 0.69 | 11.23 ± 1.05 | F(1.19,8.34) = 4.942, p=.05, η^2^ =.414 |
|  |  | LnCap | 9.22 ± 0.28 | 9.31 ± 0.41 | 9.37 ± 0.48 | F(2,22) = .047, p=.95, η^2^ =.004 |
|  |  | Pc3 | 19.38 ± 3.22 | 13.79 ± 1.34 | 12.90 ± 1.52 | F(2,6) = 3.110, p=.11, η^2^ =.509 |
| Velocity | FW | Du145 | 347.36 ± 159.7 | 303.15 ± 32.97 | 272.54 ± 27.19 | F(1.08,14.14) = .150, p=.72, η^2^ =.011 |
| (µm/s) |  | LnCap | 175.86 ± 21.47 | 172.29 ± 15.92 | - | F(1,4) =.013, p=.91, η^2^ =.003 |
|  |  | Pc3 | 165.0 ± 14.19 | 204.96 ± 31.02 | 177.12 ± 32.12 | F(2,6) = 1.191, p=.36, η^2^ =.284 |
|  | DSC | Du145 | 117.27 ± 13.39 | 104.99 ± 12.50 | 104.72 ± 17.90 | F(2,14) = .264, p=.77, η^2^ =.036 |
|  |  | LnCap | 139.71 ± 9.26 | 111.11 ± 7.34 | 112.84 ± 10.41 | F(2,22) = 3.583, p=.04, η^2^ =.246 |
|  |  | Pc3 | 106.80 ± 30.68 | 115.34 ± 16.71 | 137.06 ± 15.96 | F(2,6) = .746, p=.51, η^2^ =.199 |
| Blood flow rate | FW | Du145 | 38.75 ± 8.05 | 44.32 ± 11.17 | 44.74 ± 4.73 | F(2,26) = .145, p=.86, η^2^ =.011 |
| (µm^3^/s) × 10^3^ |  | LnCap | 31.35 ± 8.80 | 32.65 ± 2.94 | - | F(1,4) =.020, p=.89, η^2^ =.005 |
|  |  | Pc3 | 33.83 ± 11.24 | 53.99 ± 25.66 | 53.15 ± 15.16 | F(2,6) = .355, p=.71, η^2^ =.106 |
|  | DSC | Du145 | 9.02 ± 1.85 | 9.17 ± 2.83 | 11.75 ± 4.50 | F(2,14) = .769, p=.48, η^2^ =0.99 |
|  |  | LnCap | 11.1 ± 0.73 | 8.78 ± 0.92 | 8.35 ± 0.91 | F(2,22) = 2.822, p=.08, η^2^ =.204 |
|  |  | Pc3 | 33.22 ± 11.6.1 | 19.96 ± 2.53 | 20.71 ± 6.17 | F(2,6) = .907, p=.45, η^2^ =.232 |
| Vessel density | FW | Du145 | 110.43 ± 6.74 | 116.25 ± 9.75 | 126.04 ± 5.94 | F(2,26) = 1.118, p=.34, η^2^ =.079 |
| (cm/cm^2^) |  | LnCap | 102.22 ± 7.93 | 120.53 ± 11.59 | - | F(1,4) = 2.706, p=.17, η^2^ =404 |
|  |  | Pc3 | 75.21 ± 20.03 | 142.19 ± 15.51 | 94.63 ± 13.52 | F(2,6) = 6.503, p=.03, η^2^ =.684 |
|  | DSC | Du145 | 179.52 ± 20.01 | 216.68 ± 21.97 | 123.44 ± 24.40 | F(2,14) = 5.536, p=.01, η^2^ =.442 |
|  |  | LnCap | 180.29 ± 13.33 | 195.62 ± 10.42 | 168.79 ± 20.66 | F(1.28,14.13) = .731, p=.49, η^2^ =.062 |
|  |  | Pc3 | 61.36 ± 18.22 | 100.9 ± 19.42 | 184.01 ± 44.99 | F(2,6) = 5.308, p=.04, η^2^ =.639 |
| Tissue perfusion | FW | Du145 | 65.76 ± 14.40 | 60.99 ± 13.30 | 67.66 ± 9.74 | F(2,26) = .067, p=.93, η^2^ =.005 |
| (ml/cm^2^/s) × 10^-5^ |  | LnCap | 40.41 ± 1.04 | 66.04 ± 3.72***** | - | F(1,4) =7.973, p=0.4, η^2^ =.666 |
|  |  | Pc3 | 53.11 ± 13.77 | 88.61 ± 43.79 | 48.3 ± 11.77 | F(2,6) =.363, p=.710, η^2^ =.108 |
|  | DSC | Du145 | 14.87 ± 1.71 | 19.20 ± 5.28 | 11.44 ± 2.09 | F(1.18,8.25) =1.660, p=.22, η^2^ =.192 |
|  |  | LnCap | 17.93 ± 1.19 | 18.63 ± 2.03 | 13.19 ± 1.34 | F(2,22) =3.150, p=.06, η^2^ =.223 |
|  |  | Pc3 | 13.58 ± 3.55 | 23.87 ± 6.32 | 31.93 ± 3.00 | F(2,6) =3.168, p=.11, η^2^ =.514 |
| Permeability | FW | Du145 | 6.04 ± 0.87 | 5.51 ± 0.54 | 7.04 ± 1.15 | F(2,10) =.647, p=.53, η^2^ =.061 |
| (cm/s) × 10^-3^ |  | LnCap | 6.33 ± 0.87 | 6.54 ± 1.05 | - | F(1,4) =.018, p=.90, η^2^ =.004 |
|  |  | Pc3 | 7.11 ± 1.12 | 7.9 ± 2.63 | 9.23 ± 2.50 | F(2,4) =.243, p=.79, η^2^ =.108 |
|  | DSC | Du145 | 4.58 ± 0.63 | 3.92 ± 0.63 | 4.07 ± 0.50 | F(1.03,4.14) =.558, p=.59, η^2^ =.122 |
|  |  | LnCap | 3.77 ± 0.27 | 4.34 ± 0.53 | 3.80 ± 0.21 | F(2,20) =1.215, p=.31, η^2^ =.108 |
|  |  | Pc3 | 4.11 ± 0.41 | 4.54 ± 0.37 | 4.90 ± 0.47 | F(1,5) = .043, p=.84, η^2^ =0.09 |

The data are expressed as the mean (± standard deviation).

* Statistically significant (p < 0.05) differences between days 7 and 14, and days 14 and 21.

****** Statistically significant (p < 0.05) differences between days 7 and 21.
